# Prolonged Cholestatic Hepatitis A With Transient Epstein-Barr Virus IgM Reactivity and Marked Hyperferritinemia in an HFE H63D Heterozygote

**DOI:** 10.7759/cureus.101467

**Published:** 2026-01-13

**Authors:** Sara R Silva, Filipe Dias, Cláudia Ribeiro, Fátima Augusto, Cátia Albino

**Affiliations:** 1 Internal Medicine, Hospital do Litoral Alentejano, Santiago do Cacém, PRT; 2 Internal Medicine, Unidade Local de Saúde do Litoral Alentejano, Santiago do Cacém, PRT; 3 Gastroenterology and Hepatology, Unidade Local de Saúde do Litoral Alentejano, Hospital da Luz Setúbal, Santiago do Cacém, PRT

**Keywords:** cholestasis, epstein–barr virus, epstein–barr virus serology pitfalls, hemochromatosis, hepatitis a, hepatitis a biphasic course, hfe h63d, hyperferritinemia

## Abstract

Hepatitis A virus (HAV) infection is usually self-limited and does not progress to chronic liver disease. However, atypical courses such as prolonged cholestatic hepatitis may occur in adults, posing diagnostic and therapeutic challenges. We report the case of a 55-year-old man with hypertension, obesity, and known hepatic steatosis who presented with jaundice, choluria, acholic stools, fatigue, epigastric pain, and nausea. Laboratory evaluation revealed a mixed hepatocellular-cholestatic pattern with predominantly direct hyperbilirubinemia. Acute HAV infection was confirmed (anti-HAV IgM positive), and alternative causes were excluded. Imaging showed no biliary obstruction. Epstein-Barr virus (EBV) serology obtained during the first admission was consistent with past infection (viral capsid antigen (VCA) IgG positive, VCA IgM negative, and EBV nuclear antigen-1 (EBNA-1) IgG positive). The patient improved with supportive care and was discharged after one week.

He was readmitted one week later with clinical relapse and severe hyperbilirubinemia (total bilirubin 25.3 mg/dL). Given a household contact with a mononucleosis-like illness, repeat EBV serology showed weak/low-level VCA IgM reactivity (12.1 UA/mL) with persistent VCA IgG positivity. However, subsequent reassessment results returned to VCA IgM negativity with persistent VCA IgG and EBNA-1 IgG positivity, supporting remote EBV infection and suggesting non-specific IgM reactivity (or, less likely, reactivation) rather than primary acute EBV infection. EBV DNA PCR was not pursued due to low clinical and serologic suspicion of active infection and the subsequent resolving course. A genetic study requested during the first admission revealed HFE H63D heterozygosity, with a ferritin level >11,000 ng/mL and transferrin saturation of >80%. The patient improved with ursodeoxycholic acid (UDCA) and individualized iron management. Corticosteroids were not used, given progressive improvement with supportive care and UDCA. Follow-up quantitative liver MRI showed only mild iron overload (43 µmol/g), supporting an acute-phase/inflammatory iron contribution during severe hepatitis, and FibroScan® revealed mild fibrosis (5.3 kPa). This case highlights the importance of stepwise reassessment in prolonged cholestatic HAV, the pitfalls of interpreting transient EBV VCA IgM reactivity in a patient with serology consistent with prior EBV infection, and careful interpretation of marked hyperferritinemia in HFE H63D heterozygotes.

## Introduction

Hepatitis A virus (HAV) is a common cause of acute viral hepatitis and, in most people, it runs a self-limited course that settles within a few weeks with supportive care [1]. In adults, however, the presentation can be less straightforward. Cholestatic forms and prolonged or relapsing courses are well described, and they can be clinically frustrating [2]. Cholestatic hepatitis refers to an acute hepatitis presentation in which jaundice and cholestatic dysfunction (predominantly conjugated hyperbilirubinemia, often with pruritus) persist beyond the expected timeframe for typical acute hepatitis, sometimes for weeks to months [2]. It has been reported in roughly 10%-20% of acute HAV infections (and more sporadically with other viral hepatitis) [1,2]. When this happens, patients may remain jaundiced and intensely pruritic, often requiring longer admissions and repeated investigations, with an ongoing uncertainty about the best treatment approach [2].

Part of the difficulty is that the exact mechanisms of liver injury in hepatitis A are still not fully understood, even after decades of research [3]. As a result, there is no widely accepted, standardized therapeutic pathway, particularly for cholestatic or prolonged/relapsing disease. Different strategies have been proposed, but agreement on what works best is limited. A systematic review that focused on treatment options for prolonged cholestatic HAV infection highlighted this variability across 164 studies [4]. Only two case reports described benefit with ursodeoxycholic acid (UDCA), using doses between 10 and 30 mg/kg/day [4]. Corticosteroids were the most frequently reported intervention, appearing in 21 studies, usually with prednisolone doses between 30 and 60 mg/day [4]. For patients who did not respond to these measures, a small number of reports described “rescue” options: nasobiliary drain placement in two patients and plasma exchange in three [4]. Overall, the authors proposed a pragmatic, stepwise approach: if symptoms persist despite UDCA or cholestyramine, a short trial of corticosteroids may be reasonable, which also supports the idea that an immune-mediated component may be involved. In steroid-refractory cases, nasobiliary drainage or plasma exchange can be considered, but only after carefully weighing risks and potential benefits for the individual patient [4].

When HAV behaves in a complicated or unusually prolonged way, it is also important to consider whether additional factors are contributing. Coexisting liver disease can clearly prolong recovery. Conditions such as hereditary hemochromatosis (HH), hepatic steatosis, or other viral infections with hepatic involvement, such as Epstein-Barr virus (EBV), may complicate the clinical picture and delay resolution [5,6].

Regarding EBV, it is relevant in this setting because it can cause hepatitis with cholestatic features and because serologic interpretation may be confounded by non-specific or transient IgM reactivity during systemic inflammatory illnesses [6].

HH is an inherited iron overload disorder caused by increased intestinal iron absorption due to inadequate hepcidin activity [5,7]. It is among the most common genetic conditions in individuals of Northern European ancestry [8]. However, ferritin is an acute-phase reactant and can rise markedly during inflammatory liver injury, which may obscure the distinction between inflammatory hyperferritinemia and true iron overload [5,7]. Consequently, very high ferritin levels during acute hepatitis can complicate interpretation and management decisions, particularly when iron-related genetic variants are identified, making it important to differentiate acute-phase changes from clinically relevant underlying iron overload [5,7].

We describe a case of prolonged cholestatic hepatitis A in an adult with hepatic steatosis and extreme hyperferritinemia in an HFE H63D heterozygote, highlighting the importance of stepwise reassessment in biphasic/prolonged HAV and the pitfalls of interpreting transient EBV IgM reactivity in a patient with serology consistent with prior EBV infection.

## Case presentation

A 55-year-old man with hypertension (on nebivolol 5 mg/day and candesartan/hydrochlorothiazide 16/12.5 mg/day), obesity, and known hepatic steatosis presented to the emergency department with jaundice, dark urine, pale stools, fatigue, epigastric pain, and nausea. He had no known drug allergies.

**Figure 1:**
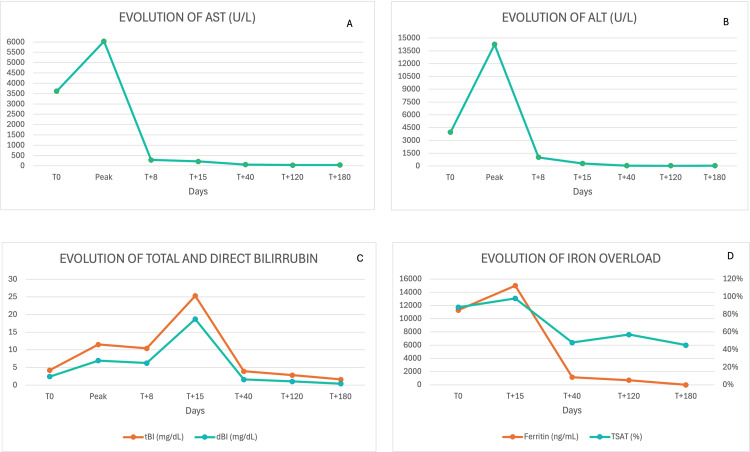
Six-month evolution of AST (A), ALT (B), total and direct bilirubin (C), and iron indices (D) during hepatitis A virus (HAV) infection A biphasic pattern is observed, with peak aminotransferases during the first admission and peak cholestasis and iron indices during relapse. AST, aspartate aminotransferase; ALT, alanine aminotransferase; tBI, total bilirubin; dBI, direct bilirubin; TSAT, transferrin saturation

**Figure 2:**
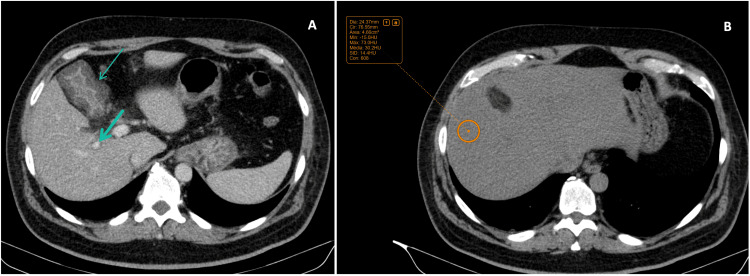
Abdominopelvic CT at first admission (A) Marked gallbladder wall thickening (thin arrow), measuring approximately 13 mm, with pericholecystic fat stranding, without luminal distension or radiopaque cholelithiasis and periportal low attenuation/halo, compatible with periportal edema (thick arrow), both non-specific findings that may be seen in acute hepatitis [11]. There was no evident intrahepatic biliary ductal dilatation. (B) Diffuse decrease in hepatic parenchymal attenuation, consistent with hepatic steatosis (mean attenuation ~30 HU).

**Figure 3:**
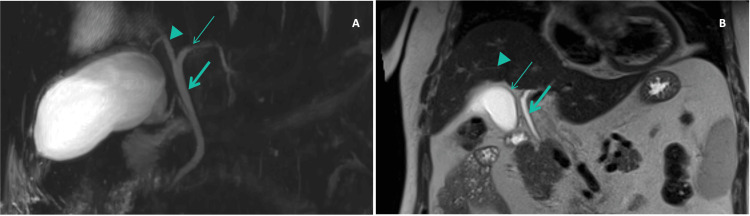
Magnetic resonance cholangiopancreatography (MRCP) performed during the first admission, a few days after the abdominopelvic CT (A) Coronal T2-weighted HASTE (half-Fourier acquisition single-shot turbo spin-echo) MRCP image showing no dilatation of the main bile ducts, with visualization of the common hepatic duct (thick arrow), left hepatic duct (thin arrow), and right hepatic duct (arrowhead). (B) Heavily T2-weighted cholangiographic MRCP image demonstrating no dilatation of the extrahepatic bile ducts (thick arrow) or intrahepatic bile ducts (arrowhead). There is also marked improvement in the gallbladder wall thickening (thin arrow) previously seen on CT, despite persistent cholestasis.

Initial laboratory evaluation (Table 1; Figure 1) showed a mixed hepatocellular-cholestatic pattern, with predominant hepatocellular injury and predominantly direct (conjugated) hyperbilirubinemia (aspartate aminotransferase (AST) 3622 U/L; alanine aminotransferase (ALT) 3963 U/L; gamma-glutamyl transferase (GGT) 92 U/L; alkaline phosphatase (ALP) 86 U/L; total bilirubin 4.20 mg/dL, direct bilirubin 2.42 mg/dL). The etiological workup confirmed acute HAV infection (anti-HAV IgM positive). Serologies for hepatitis B virus (HBV), hepatitis C virus (HCV), hepatitis E virus (HEV), and HIV were negative. Cytomegalovirus (CMV) testing did not meet criteria for acute infection. EBV serology was consistent with past infection (viral capsid antigen (VCA) IgG positive, VCA IgM negative, and EBV nuclear antigen-1 (EBNA-1) IgG positive), with no serologic pattern suggestive of primary acute EBV infection. Given clinical deterioration and worsening biochemical abnormalities, along with evidence of mild hepatic dysfunction, additional tests were ordered. Abdominal ultrasound showed no biliary ductal dilation and revealed hepatic steatosis, splenomegaly, and minimal ascites. An abdominopelvic CT scan (Figure 2) showed marked gallbladder wall thickening (~13 mm) with pericholecystic fat stranding (Figure 2A), without gallbladder distension or radiopaque cholelithiasis; this gallbladder wall thickening was considered a non-specific finding that may be related to acute hepatitis. The CT also demonstrated a periportal low-attenuation halo compatible with edema, no evident intrahepatic biliary ductal dilatation, and diffuse low hepatic attenuation (~30 HU) consistent with steatosis (Figure 2B). Magnetic resonance cholangiopancreatography (MRCP) excluded biliary obstruction and showed no intrahepatic or extrahepatic bile duct dilatation (Figure 3), with interval improvement of the gallbladder wall thickening previously seen on CT.

**Table 1:** Six-month evolution of the patient's blood test results during hepatitis A virus (HAV) infection AST, aspartate aminotransferase; ALT, alanine aminotransferase; GGT, gamma-glutamyl transferase; ALP, alkaline phosphatase; tBI, total bilirubin; dBI, direct bilirubin; PT, prothrombin time; TSAT, transferrin saturation Normal reference values are according to references [9,10].

Blood work analysis	At first admission (T0 days)	Peak values at first admission (peak)	At first discharge (T+8 days)	At early outpatient reassessment/second admission (T+15 days)	At second discharge (T+40 days)	At 4-month follow-up (T+120 days)	At 6-month follow-up (T+180 days)	Normal reference values
AST (U/L)	3622	6035	289	215	63	40	45	<35 U/L
ALT (U/L)	3963	14249	1014	289	37	27	31	<45 U/L
GGT (U/L)	92	99	76	73	25	18	17	7-60 U/L
ALP (U/L)	86	110	90	133	96	87	86	40-129 U/L
tBI (mg/dL)	4.20	11.50	10.40	25.30	3.90	2.80	1.60	0.3-1.2 mg/dL
dBI (mg/dL)	2.42	6.95	6.25	18.69	1.59	1.03	0.40	0.1-0.3 mg/dL
PT (seconds)	19.5	31.5	16.5	15	13.08	14.5	13	10-14 seconds
Albumin (g/dL)	3.7	2.4	2.4	2.9	3.0	3.5	4.0	>3.5 g/dL
Ferritin (ng/mL)	11291	NA	NA	>15000	1152	691	288.6	<300 ng/mL
TSAT (%)	88%	NA	NA	98%	48%	57%	45%	<50%

The patient was managed with supportive care (intravenous fluids and analgesia). He improved clinically and biochemically (Table 1; Figure 1), with declining aminotransferases and total bilirubin remaining stable at approximately 10 mg/dL. He was discharged after one week.

One week later, at early outpatient reassessment, he reported worsening jaundice, recurrence of epigastric pain, nausea, and poor oral intake. Laboratory tests revealed recurrent mixed hepatocellular-cholestatic injury, with severe hyperbilirubinemia (25.30 mg/dL), predominantly direct (direct bilirubin 18.69 mg/dL), prompting readmission. This evolution supported a prolonged cholestatic phase with clinical relapse rather than persistent hepatocellular necroinflammation (Table 1; Figure 1C). Because his daughter had developed symptoms compatible with infectious mononucleosis, repeat infectious testing was performed. Anti-HAV IgM remained positive. Repeat EBV serology showed weak/low-level VCA IgM reactivity (12.1 UA/mL) with persistent VCA IgG positivity. Given that EBNA-1 IgG was already positive during the first admission, this profile was interpreted cautiously as most consistent with remote EBV infection with possible non-specific IgM reactivity (and less likely reactivation), rather than primary acute EBV infection.

A genetic test requested during the first admission showed HFE H63D heterozygosity. Iron studies demonstrated ferritin levels at >11,000 ng/mL and transferrin saturation of >80%. Abdominal MRI performed during the second admission suggested hepatic iron overload, without quantification at that stage. The patient was started on UDCA 1,200 mg/day and underwent therapeutic phlebotomy (450 mL). On subsequent reassessment, EBV serology reverted to VCA IgM negativity with persistent VCA IgG and EBNA-1 IgG positivity, further supporting remote EBV infection rather than ongoing acute EBV hepatitis. EBV DNA PCR was not performed because there was no clinical syndrome suggestive of active EBV infection, and the overall serologic profile remained consistent with remote infection, with subsequent spontaneous reversion of VCA IgM to negativity. Over the following three weeks, he showed progressive clinical and laboratory improvement, allowing discharge. Corticosteroids were not initiated because symptoms and cholestasis began to improve with supportive care/UDCA, and given the limited evidence base and potential adverse effects in this context.

**Figure 4:**
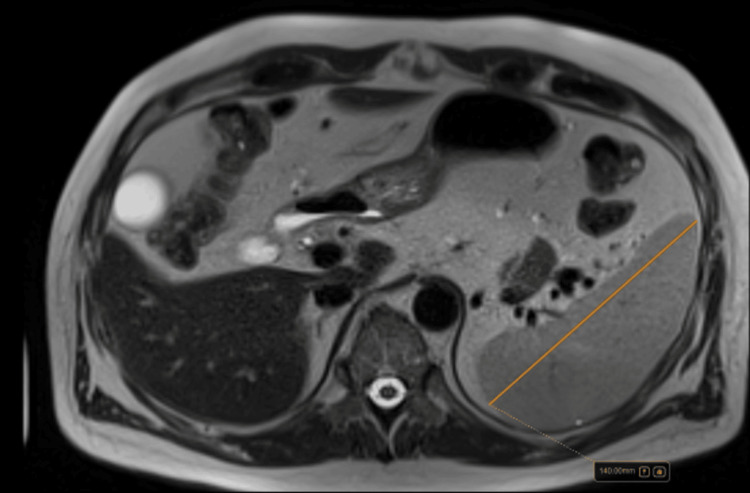
Follow-up MRI (axial T2-weighted image) demonstrating splenomegaly (maximum craniocaudal length ~140 mm)

At the Internal Medicine clinic at the four-month follow-up, quantitative liver MRI showed mild hepatic iron overload (43 µmol/g) without significant steatosis, with persistent splenomegaly (140 mm) (Figure 4). FibroScan® showed minimal steatosis (ultrasound-derived fat fraction, or UDFF, 4.8%) and mild fibrosis (5.3 kPa, F1-F2). Liver tests (Table 1; Figure 1) continued to normalize (total bilirubin 2.8 mg/dL), and the ferritin level decreased to <700 ng/mL, with transferrin saturation around 58%. Because of persistent symptoms (headache and abdominal discomfort) and elevated iron indices, an additional 250 mL phlebotomy was performed with immunohematology support, leading to symptomatic improvement and a current transferrin saturation of 45%. The patient remains under follow-up for metabolic dysfunction-associated steatotic liver disease, cardiovascular risk control, and surveillance of iron indices. He was stable at the six-month follow-up. A timeline case summary is provided in Table 2.

**Table 2:** Case summary HAV, hepatitis A virus; UDCA, ursodeoxycholic acid

Timeline case summary	
Day 0 (first admission)	Acute HAV confirmed with predominant hepatocellular injury
Day 8	Discharge after clinical improvement
Day 15 (readmission)	Relapse with severe cholestasis and marked direct hyperbilirubinemia
Subsequent weeks	Initiation of UDCA and individualized iron management with progressive recovery (Table 1; Figures 1A-1D)
Day 40	Discharge after clinical improvement
At 4-month follow-up (Day 120)	Liver MRI showed mild hepatic iron overload; FibroScan® showed minimal steatosis and mild fibrosis; liver tests and ferritin levels continued to normalize (Table 1; Figures 1A–1D); individualized iron management
At 6-month follow-up (Day 180)	Patient currently stable (Table 1; Figures 1A-1D)

## Discussion

Cholestatic hepatitis A is an uncommon but well-recognized clinical variant, typically marked by prolonged jaundice and pruritus, conjugated hyperbilirubinemia, and elevation of cholestatic liver enzymes [2,12,13]. Management is primarily supportive. Ursodeoxycholic acid is frequently used for symptom control, and a short course of corticosteroids may be considered in carefully selected patients with persistent, refractory symptoms, once alternative causes of prolonged cholestasis have been excluded [4,14,15].

This case highlights the value of reassessment when the clinical course deviates from the expected evolution of acute HAV infection. EBV serology required careful interpretation. The initial EBV profile was already consistent with past infection (VCA IgG positive with VCA IgM negative and EBNA-1 IgG positive), which argues strongly against primary acute EBV infection as a driver of the cholestatic course. At relapse, a household contact with a mononucleosis-like illness supported repeating the infectious workup; however, repeat testing showed only weak/low-level VCA IgM reactivity (12.1 UA/mL) with persistent VCA IgG positivity. In a patient with established EBNA-1 IgG positivity, this pattern is most consistent with remote EBV infection with possible non-specific IgM reactivity (and less likely reactivation), rather than primary EBV hepatitis. Subsequent reassessment with VCA IgM negativity and persistent VCA IgG/EBNA-1 IgG further supported this interpretation. Therefore, EBV is best viewed here as a potential confounder in serologic interpretation rather than a proven contributor to the biphasic/prolonged phenotype; attributing causality would require additional evidence of active replication (e.g., EBV DNA by PCR) in the appropriate clinical context [6].

Marked hyperferritinemia with high transferrin saturation in an HFE H63D heterozygote also warrants cautious interpretation, as this genotype alone rarely results in clinically significant iron overload [5,7,8]. Ferritin is a robust acute-phase reactant and can rise substantially in acute viral hepatitis and cholestasis; transferrin saturation may likewise be transiently elevated in the setting of hepatocellular injury [9,10]. In this patient, subsequent quantitative MRI demonstrated only mild hepatic iron overload, and non-invasive fibrosis assessment did not suggest advanced liver disease, supporting an individualized approach [5,8]. Therapeutic phlebotomy was therefore considered and selectively performed based on symptoms and persistently abnormal iron indices during follow-up, while acknowledging that subsequent quantitative MRI showed only mild hepatic iron overload, supporting a substantial acute-phase contribution during severe hepatitis. This underscores the importance of reserving the term “hepatic iron overload” for imaging-confirmed deposition and describing acute-phase abnormalities as “marked hyperferritinemia with elevated transferrin saturation” when inflammation/hepatocellular injury is the likely driver [5,8].

Finally, this case also reinforces the ongoing public health importance of hepatitis A vaccination. Despite the declining incidence in many high-income countries, global travel, migration, and interconnected food supply chains continue to facilitate outbreaks, underscoring the need for sustained preventive strategies [1].

## Conclusions

Prolonged cholestatic hepatitis A should be considered in adults presenting with persistent cholestatic jaundice. Clinical relapse or an unexpectedly protracted course should prompt repeat etiological evaluation and careful review of alternative contributors. EBV serology may be misleading during acute inflammatory illnesses; in patients with serology consistent with prior EBV infection, transient low-level VCA IgM reactivity should be interpreted cautiously and does not, by itself, establish acute EBV hepatitis. In HFE H63D heterozygotes with marked hyperferritinemia, quantitative MRI and non-invasive fibrosis assessment are essential to guide management and distinguish acute-phase changes from clinically relevant iron overload. In this case, UDCA provided symptomatic benefit, and the clinical course was favorable with stepwise reassessment and individualized iron management.
